# Humans but Not Chimpanzees Vary Face-Scanning Patterns Depending on Contexts during Action Observation

**DOI:** 10.1371/journal.pone.0139989

**Published:** 2015-11-04

**Authors:** Masako Myowa-Yamakoshi, Chisato Yoshida, Satoshi Hirata

**Affiliations:** 1 Graduate School of Education, Kyoto University, Yoshida-honmachi, Sakyo, Kyoto, 606–8501, Japan; 2 ERATO, Japan Science and Technology Agency, 413, Main Research Bldg., RIKEN, 2–1 Hirosawa, Wako, Saitama, 351–0198, Japan; 3 Kumamoto Sanctuary, Kyoto University, Uki, Kumamoto, 869–3201, Japan; Centre for Coevolution of Biology & Culture, University of Durham, UNITED KINGDOM

## Abstract

Human and nonhuman primates comprehend the actions of other individuals by detecting social cues, including others’ goal-directed motor actions and faces. However, little is known about how this information is integrated with action understanding. Here, we present the ontogenetic and evolutionary foundations of this capacity by comparing face-scanning patterns of chimpanzees and humans as they viewed goal-directed human actions within contexts that differ in whether or not the predicted goal is achieved. Human adults and children attend to the actor’s face during action sequences, and this tendency is particularly pronounced in adults when observing that the predicted goal is not achieved. Chimpanzees rarely attend to the actor’s face during the goal-directed action, regardless of whether the predicted action goal is achieved or not. These results suggest that in humans, but not chimpanzees, attention to actor’s faces conveying referential information toward the target object indicates the process of observers making inferences about the intentionality of an action. Furthermore, this remarkable predisposition to observe others’ actions by integrating the prediction of action goals and the actor’s intention is developmentally acquired.

## Introduction

Human and nonhuman primates are social beings who live in highly cohesive groups. This primate group living may shape sensitivity to subtle social cues. For example, understanding other individuals’ actions is crucial for anticipating their future actions and appropriately adjusting one’s own behaviour. Both monkeys and humans have common neural mechanisms underlying the capacity to predict and understand the motor goals of others’ actions, which are called mirror neurons (e.g., [1–7]). It has been argued that the mirror neuron system automatically and pre-reflexively matches action perception and action execution [3,4] (e.g., the neurons that fire when I grasp a cup of water are similar to those that fire when I watch another person grasp a cup). However, several researchers have emphasized the differences in mirror system function between monkeys and humans. For example, the vast majority of monkey mirror neurons are responsive to actions-on-objects, whereas human mirror activation occurs in response to both intransitive meaningless gestures and actions-on-objects [8–10] (regarding a cross-species comparison of mirror system circuity in monkeys, chimpanzees, and humans, see [11]).

Faces also provide crucial information about individuals’ social lives. By detecting information expressed in the faces of other individuals, such as gaze direction and emotional facial expressions, individuals are able to rapidly identify and respond to dynamic changes in their environment [12]. Whilst research investigating the roles of motor actions and facial information have proved valuable for increasing our knowledge about action understanding, to date the majority of these studies have investigated characteristics of motor actions and facial information independently. For example, the ability to understand others’ actions has been primarily studied by only focusing on transitive or goal-related motor acts, such as reaching, grasping, and manipulating an object (e.g., [13–16]). It is therefore currently unclear how the information regarding *both* goal-directed motor acts and actors' faces is integrated when understanding actions, despite the fact that both motor acts and others’ faces are observed as daily events unfold [17]. Here, using eye-tracking technology, we investigated time-series changes in scanning patterns while observing object-related actions, with a particular focus on the actor’s face.

Mirror neurons, which are responsive to actions-on-objects, may underlie action recognition in both monkeys and humans. However, humans also appear to have a predisposition to observe goal-directed actions by integrating information from the actor’s face. Previous studies have demonstrated that both humans and nonhuman primates rationally predict and understand the object-related actions of other individuals [18–20]. However, our own previous study demonstrated distinct differences between the scanning patterns of humans and chimpanzees when viewing goal-directed actions ^20^. More specifically, captive chimpanzees who were familiar with humans primarily attended to information regarding motor acts, including hand movements and manipulated objects; however, they rarely looked at the actor’s face, regardless of whether the actor was a chimpanzee or a human. By contrast, humans (both adults and infants) attended to others’ faces to a greater extent than chimpanzees.

It is likely that these two species differ with respect to when and why they refer to faces when coding an actor’s goal-directed actions. We assume that, by scanning the actor’s face, humans make ‘explicit’ or active inferences concerning whether an action is likely to have been performed intentionally. For example, facial expressions of frustration or disappointment might convey that an agent's goal had been unsuccessful or that the outcome was accidental. Therefore, when predicted motor goals are not completed, humans in particular devote substantially greater attention to the actor’s face to seek additional referential information.

Our first aim was to investigate the timing of facial references along with the process of encoding the goal-directedness of an action in humans and chimpanzees. We compared the two species’ time-series scanning patterns of actions in the following two contexts: when a possible goal was achieved and when it was not (Experiments 1 and 2). We predicted that when the possible goal was not achieved, humans would search for additional referential information from the actor. By contrast, assuming that chimpanzees understand goal-directed actions primarily based on the physical characteristics of the environment (i.e., manipulated objects), no clear differences may be evident in chimpanzees’ facial scanning patterns between the two action contexts. We show that humans do increase their attention to the actor’s face when they observe that the predicted goal is not completed whereas chimpanzees do not attend to faces even when the actor’s predicted action goal is not completed.

The second aim of the current study was to investigate the human ontogeny of the ability to use referential information for understanding others’ actions. When do humans first present evidence of adult-like social inferences? How does this ability develop? We examined the developmental changes in referential face-scanning patterns for faces when coding an actor’s goal-directed actions by comparing 12-month-old infants who do not yet use explicit language and 3.5-year-old children who use explicit language. (Experiment 3).

## Experiment 1: Facial Scanning Patterns for Object-Related Actions in Humans and Chimpanzees

### Materials and Methods

#### Participants

A total of 14 adults (seven males, mean age = 23.8 years, s. d. = 2.9 years) and six captive chimpanzees (*Pan troglodytes*: two males, 6–16 years) participated in Experiment 1. The two males (both 16 years old) and four females (15, 15, 12 and 6 years old) lived as a group. The chimpanzees were cared for at the Great Ape Research Institute, Hayashibara Biomedical Laboratories, Inc. All the chimpanzees were familiar with humans and had previously participated in several behavioural cognitive tasks, including tool use procedures, sequential learning paradigms using touch screens, and eye-tracking measures [20, 21]. The chimpanzees spent several hours each day indoors interacting with humans for study or husbandry purposes, which included play activities using materials available in human environments such as hats, glasses, gloves, socks, clothes, watches, pens, books, papers, various types of containers (cups, bottles, buckets, etc.) and so forth.

#### Ethics statement

All the experiments in this study were approved by the Ethical Committee of Kyoto University, Kokoro-Unit. This study was conducted in accordance with the standards specified in the 1964 Declaration of Helsinki. The care and use of chimpanzees adhered to the guidelines established by the Primate Society of Japan. This study was approved by the Animal Welfare and Animal Care Committee of the Hayashibara Biochemical Laboratories. We have obtained consent from the model actor to her image being used in the figures and video files (in Supporting Information) of the present paper.

#### Apparatus and stimuli

A Tobii (Stockholm, Sweden) T60 Eye Tracker integrated with a 17-inch TFT monitor was used to present the stimuli and record eye movements using image-processing algorithms (60 Hz; Tobii Studio 2.1.12, Tobii Technology). This eye tracker could record eye gazes from both humans and chimpanzees without using body mounting devices such as glasses. The participants were seated approximately 60-cm from the monitor and no barrier was involved. The stimulus presentation and recording were controlled via a computer (Dell T7500 for human adults, Dell M4400 for chimpanzees) with Tobii Studio software. The entire video subtended 21.6°×16.2° of the visual angle. Prior to the video presentation, small animation videos were shown to the participants to direct their attention to the monitor.

Test stimuli were videos depicting an unfamiliar female human adult actor seated in front of a table in a well-lit room, who either poured some juice from a bottle into a clear glass cup (*Congruent* action) or poured some juice onto the table top (*Incongruent* action). Both videos lasted 14.0 s, and were approximately matched in relation to temporal features (i.e., the velocity of the actor’s hand movements and the timing when the actor made the pouring motion; specific details of timing can be found in the Methods and the velocity profiles for the actor’s right hand during these videos are shown in S2 Fig). Both the human and chimpanzee participants were familiar with this type of goal-directed action and were capable of performing the action by themselves. For example, the chimpanzees received juice as a part of their meal in the morning (juice was on their breakfast menu on average 3–4 times per week) and occasionally in the daytime. The chimpanzees watched familiar human staff pour the juice in front of them from a bottle into a cup, and they drank from the cup. In addition, various types of containers were provided in their enclosures for enrichment purposes, and all the chimpanzees in the present study had been observed, multiple times, engaging in pouring liquid materials from one container to another (e.g., pouring pure water from a bottle to a cup) during their free activities by using these enrichment materials.

For chimpanzees, the calibration errors were estimated prior to testing, and the average rate of error across the participants was 0.40° (s.d. = 0.38°) of the chimpanzees’ visual angle [21]. We did not precisely measure the calibration errors for the human groups given the accumulated knowledge regarding the validity of data collection using the device we employed [13, 14, 20, 22]. However, the errors can be estimated to be within the range of 1° of the visual angle of most of our participants, based on their fixation data from the stimulus used for attention-getting. One degree of visual angle is larger than the difference between the outline of each feature (i.e., face, cup or container, and trajectory of the manipulated object) and that of the respective area of interest AOI (see Data Analysis). Therefore, it is unlikely that calibration errors affected our analyses of gaze behaviour.

#### Procedure

Upon arrival at the laboratory, human participants were brought into the study room which was softly illuminated to make the monitor screen the most salient feature of the room. An initial calibration procedure was conducted which was completed when measures from five calibration points were obtained. This procedure was repeated until the calibration criterion was met. The participants were instructed to simply watch the video until it ended. Human participants did not receive an external reward (food or social) for their participation. Chimpanzees were brought from their enclosures into a study room which was connected to their enclosures via a passageway by calling their names. They could receive a part of their daily meals in this room, which was a daily routine procedure for study and husbandry purposes. Familiar human experimenters remained in the study room during testing. One experimenter sat beside the chimpanzee and lightly held their chins or heads during the recording. This was to ensure that the distance between their eyes and the monitor remained appropriate for testing. The calibration for each chimpanzee was achieved at the beginning of the session by presenting a brief video clip at two calibration points. This relatively small number of reference points (compared to humans) was adopted for chimpanzees because they tend to view these reference points only briefly; if a large number of calibration points are presented they tend not to look at all of them, which would result in calibration failure [15, 21]. Despite this methodological difference between humans and chimpanzees our validation session with respect to the latter’s calibration data confirmed a comparable accuracy between species. All participants were then shown the videos involving the actor. The experimenter gave food to the chimpanzees before and after the completion of the video but not while they were viewing the video. Before the video presentation, food was given if the chimpanzees sat in front of the monitor. After the video presentation, they could receive a food reward regardless of their behaviour while watching the video. The experiment relied on voluntary participation by the chimpanzees, and they were not deprived of food or water for testing. That is, they could receive their daily meals regardless of whether they participated in the study. During testing they presented no negative emotional expressions such as screaming or grimacing.

The human and chimpanzee participants were shown two repetitions of the congruent action video followed by two repetitions of the incongruent version (a session), which were separated by an interval of approximately 5 to 7 s (*Congruent*―interval―*Congruent*―interval―*Incongruent*―interval―*Incongruent*). The order of the C-C-I-I session was fixed to minimize the expectation violation effect. During the interval, an animation or video clip was presented.

#### Data analysis

Fixations were scored using a Tobii fixation filter with a threshold radius of 35 pixels. The statistical tests were calculated using SPSS 22.0 (IBM Corp.). We applied parametric tests after examining the normality of our data sample using graphical inspection of a Q–Q plot for normality and conducting a Shapiro–Wilk test. The looking time data for the first trials of the congruent and incongruent actions were used for subsequent analysis. If the total looking time on the first trial in each action condition was less than 25% of a video’s duration, the data from the second trial was used. These data were analysed using angular transformations.

**Fig 1:**
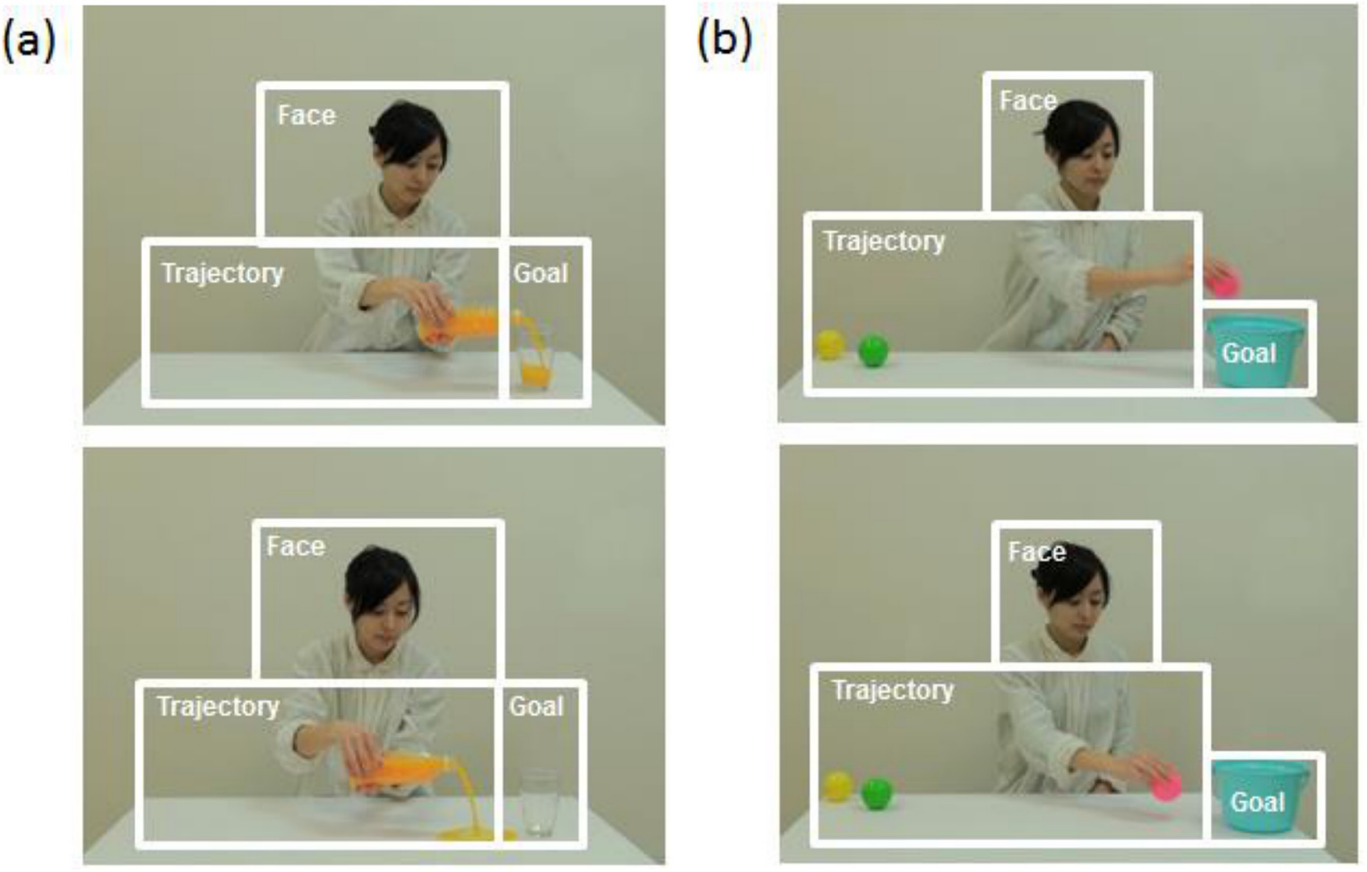
A selected scene from the video stimulus used in Experiments 1, 2, and 3 and areas of interest (AOIs) for the analyses. (a) Experiments 1 and 3: A female human adult actor sits in front of a table and pours some juice from a bottle into a clear glass cup (congruent action, upper) or pours some juice onto the floor (incongruent action, below). The videos of both the congruent and incongruent actions lasted 14.0 s. (b) Experiment 2: A female human adult actor sits at a table and places balls into a container (congruent action, upper) or places balls on the floor (incongruent action, below). The video of the congruent action lasted 13.5 s, and the video of the incongruent action lasted 13.9 s.

**Fig 2:**
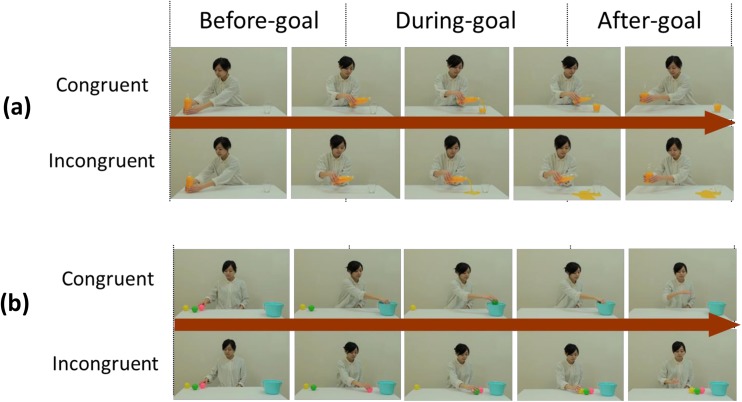
(a) Experiments 1 and 3: A female human adult actor sits in front of a table and pours juice from a bottle into a clear glass cup (congruent action, upper) or pours juice onto the table top (incongruent action, below). (b) Experiment 2: A female human adult actor sits at a table and places balls into a container (congruent action, upper) or places balls on the table top (incongruent action, below). These video stimuli were divided into the three phases of before, during, and after goal achievement. The before-goal phase began with the frame in which the manipulation of the object began and concluded with the frame depicting the onset of pouring juice or transporting the first grasped ball. The during-goal phase began with the frame depicting the onset of pouring or transporting and concluded with the frame depicting the end of the pouring or transporting action. The after-goal phase began with the frame depicting the end of the pouring or transporting action and concluded with the frame showing that the actor’s hand had returned to the location where the manipulation of the object began.

For the analysis, we defined the three AOIs for the two actions as follows: the majority of the trajectory of the moving object (Trajectory AOI), the target object (Goal AOI), and the actor’s face during manipulation (Face AOI). The initiation of goal achievement was defined as the onset of pouring juice into a cup (*Congruent* action) or onto the table top (*Incongruent* action; Fig 1A). The video stimuli were further divided into three phases as follows: before-, during-, and after-goal achievement phases (Fig 2A). The before-goal phase began with the frame at which the manipulation of the object began and concluded with the frame depicting the onset of pouring juice (3.6 s for the congruent action and 3.4 s for the incongruent action). The during-goal phase began with the frame depicting the onset of pouring and concluded with the frame depicting the end of the pouring action (7.7 s for the congruent action and 7.9 s for the incongruent action). The after-goal phase began with the frame depicting the end of pouring and concluded with the frame depicting that the actor’s hand had returned to the location at which the manipulation of the object began (4.8 s for the congruent action and 4.6 s for the incongruent action). Two-tailed paired-sample t-tests using the Bonferroni correction were used for the pairwise comparisons.

### Results

#### Preliminary analysis

Both humans and chimpanzees anticipated the goal of the action prior to the onset of pouring the juice into the cup (see S1 Text and S1A Fig). The mean durations of time human adults spent looking toward the stimuli were 8.1 s (s.d. = 0.9) for the congruent action and 8.3 s (s.d. = 1.3) for the incongruent action, whilst chimpanzee spent 5.9 s (s.d. = 2.4) for the congruent action and 4.6 s (s.d. = 2.1) for the incongruent action. The total fixation durations revealed a significant effect of group (*F*_1,18_ = 25.58, *P* < 0.001, *η* ^2^ = 0.35). Next, we compared the fixation durations for the face and object areas (i.e., Goal plus Trajectory AOIs) between humans and chimpanzees. Previous studies that presented still photographs as the test stimuli demonstrated that chimpanzees generally move their eyes more rapidly than humans [23, 24]. However, the average fixation durations did not differ between the two species (285 ms for chimpanzees, 344 ms for humans; *F*_1,18_ = 1.02, *P* = 0.33).

#### Attentional allocation to an actor’s face

We assessed when and how chimpanzees and humans referred to the actor’s face (ratio of looking time across the three AOIs combined) while viewing the goal-directed actions which consisted of the following three phases: before, during, and after goal achievement (S2 Fig).

**Fig 3:**
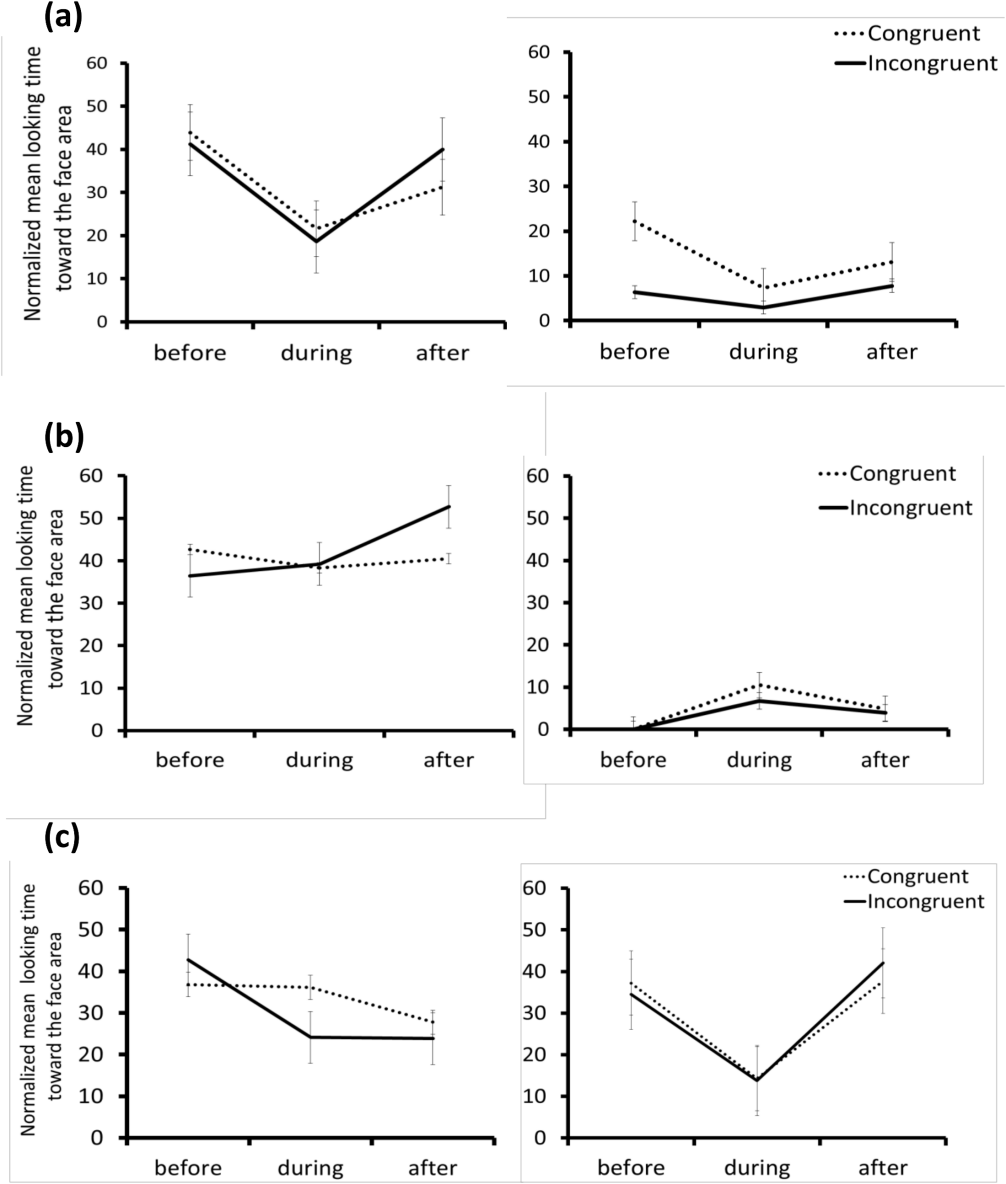
(a) Ratios of looking times towards the face area to total looking times towards the three areas combined across the three phases in Experiment 1; human adults (left) and chimpanzees (right). (b) Ratios of looking times towards the face area to total looking times towards the three areas combined across the three phases in Experiment 2; human adults (left) and chimpanzees (right). (c) Ratios of looking times towards the face area to total looking times towards the three areas combined across the three phases in Experiment 3; 12-month-old human infants (left) and 3.5-year-old human children (right). The error bars represent the standard error of the mean (SEM).

We analysed the ratio of the fixations on the actor’s face area to the total time looking toward the three areas combined. A 3 (phase: before-, during-, after-goal) × 2 (action type: congruent, incongruent) × 2 (group: humans, chimpanzees) mixed ANOVA revealed significant main effects for group (*F*_1,18_ = 15.29, *P* < 0.01, partial *η* ^2^ = 0.46) and phase (*F*_2,36_ = 7.43, *P* < 0.01, partial *η* ^2^ = 0.29). A 3 (phase) × 2 (action type) repeated-measures ANOVA for each group revealed a significant main effect of phase in humans (*F*_2,26_ = 11.24, *P* < 0.01, partial *η* ^2^ = 0.46) but not in chimpanzees (*F*_2,10_ = 1.30, *P* = 0.32, partial *η* ^2^ = 0.21). *Post hoc* testing (Bonferroni adjusted) for the human group revealed that the ratio of looking toward the face area in the before-goal phase was significantly higher than in the during-goal phase in both the congruent and incongruent conditions (*Ps* < 0.01). Importantly, only in the incongruent condition was the ratio of looking time toward the face area higher in the after-goal phase than in the during-goal phase (*P* < 0.01, Fig 3A; also see S1 and S2 Movies). The present findings suggest that in implausible contexts, humans recruit active social inferences.

In Experiment 1, two factors influenced the design of the test stimuli. First, the actions that were depicted are present in the daily experiences of both the humans and the chimpanzees participating in this study. Second, most object-related actions that have been observed in wild chimpanzees (e.g., tool-using behaviours) are aimed at obtaining food [25]. However, the possibility remains that the species differences that we observed here may be a result of the chimpanzees simply attending to the food present in the videos. To eliminate this possibility, we conducted another experiment (Experiment 2).

## Experiment 2: Facial Scanning Patterns for Non-Food-Related Actions

### Materials and Methods

#### Participants

An additional 14 human adults (seven males, mean age = 24.5 years, s.d. = 3.1 years) and the same six chimpanzees participated in Experiment 2.

#### Apparatus and stimuli

The apparatus and stimuli were nearly identical to those of Experiment 1, with the following exception. The video stimuli for Experiment 2 depicted an unfamiliar female human adult actor seated at a table placing balls into a container (*Congruent* action) or placing balls on the table top (*Incongruent* action, Fig 1B). There was no food presented in the videos. Both the human and chimpanzee participants were capable of performing this action. The actor maintained a passive face and directed her gaze towards the manipulated object as she performed the action.

#### Procedure and data analysis

The procedure and data analysis were identical to those of Experiment 1, with the following exception. In Experiment 2, the initiation of goal achievement was defined as the onset of transporting a ball into a container (*Congruent* action) or onto the table top (*Incongruent* action). As in Experiment 1, the video stimuli were further divided into three phases as follows: the before-, during-, and after-goal achievement phases (Fig 2B). The before-goal phase began with the frame at which the manipulation of the object began and concluded with the frame depicting the onset of transporting the first grasped ball (2.1 s for the congruent action and 2.5 s for the incongruent action). The during-goal phase began with the frame depicting the onset of the transporting action and concluded with the frame depicting the end of the transporting action (7.6 s for the congruent action and 8.4 s for the incongruent action). The after-goal phase began with the frame depicting the end of transporting and concluded with the frame showing that the actor’s hand had returned to the location at which the manipulation of the object began (1.7 s for the congruent action and 1.7 s for the incongruent action).

### Results

We analysed the ratio of looking time towards the face to the total looking time towards the three areas (Face, Trajectory, Goal AOIs) combined. A 3 (phase) × 2 (action type) × 2 (group) mixed ANOVA revealed significant main effects of group (*F*_1,18_ = 44.04, *P* < 0.001, partial *η* ^2^ = 0.71). A 3 (phase) × 2 (action type) repeated-measures ANOVA for each group revealed a significant interaction between phase and action type for humans (*F*_2,36_ = 4.35, *P* < 0.03, partial *η* ^2^ = 0.25), but no significant interaction was found in chimpanzees (*F*_2,10_ = 1.88, *P* = 0.20, partial *η* ^2^ = 0.27). *Post hoc* testing (Bonferroni) for the human group revealed that the ratio of looking towards the face area in the before-goal phase was significantly higher than in the during-goal phase in both the congruent and incongruent conditions (*Ps* < 0.01). Importantly, in the incongruent condition, the ratio of looking time towards the face area was higher in the after-goal phase than in the during-goal phase (*P* < 0.01, Fig 3B; also see S1 Text and S3 and S4 Movies).

Taken together, the data from Experiments 1 and 2 supported our hypothesis. Human adults significantly increased their attention to the actor’s face when they observed that the predicted goal was not completed, whereas chimpanzees did not present these facial attention patterns, regardless of whether the predicted action goal was completed or not. Given this species difference, one may subsequently question *when* does this capacity first develop in humans?

Previous studies have demonstrated that by 12–14 months of age, humans begin to use referential information (e.g., gaze direction and facial expressions) to predict the action goals of other individuals [26–28]. Infants begin to track the direction of others’ attention to specific objects or aspects of the environment as they gain the understanding that certain relationships associate these referential cues to their referents. In Experiment 3, we investigated the ontogeny of the scanning patterns in collaboration with the coding process for goal-directedness.

## Experiment 3: Developmental Changes of Facial Scanning Patterns in Humans

### Materials and Methods

#### Participants

Fifteen full-term 12-month-old human infants (8 males, mean age = 12 months and 8 days, s.d. = 8 days) and 15 3.5-year-old human children (8 males, mean age = 43 months and 6 days, s.d. = 56 days) participated in Experiment 3. An additional four 12-month-olds and one child were tested but excluded for inattentiveness (n = 5) during the sessions. All participant’s parents provided written consent according to the guidelines specified by the Ethical Committee of Kyoto University, Kokoro-Unit.

#### Apparatus and stimuli

A Tobii (Stockholm, Sweden) TX300 Eye Tracker integrated with a 23-inch TFT monitor was used to present the stimuli and record eye movements using image-processing algorithms (300 Hz; Tobii Studio 2.1.12, Tobii Technology). The stimulus presentation and recording were controlled via a computer (Dell M6600) with Tobii Studio software. The entire video subtended 21.6°×16.2° of visual angle. Prior to the video presentation, small animation videos were presented to the participants to direct their attention to the monitor. The test stimuli for Experiment 3 were identical to those of Experiment 1 (Fig 1A): videos depicting a female human adult actor sitting in front of a table who either poured some juice from a bottle into a clear glass cup (*Congruent* action) or poured some juice onto the table top (*Incongruent* action). All the children but not the infants were capable of performing this action; the infants could perform similar, albeit simpler, versions of this action, such as placing an object in one container into another container.

#### Procedure and data analysis

Upon arrival to the laboratory, infants and children were brought into the study room with their parents. The infants were placed on their parents’ laps and were seated centrally in front of the monitor. An initial calibration procedure was conducted which was completed when measures from five calibration points were obtained. This procedure was repeated until the calibration criterion was met for each infant. For the children, the same procedure was followed with the exception that they sat in a normal chair during the experiment. The analysis was identical to that of Experiment 1 (Fig 2A).

### Results

The 3.5-year-old children shifted their gaze to the goal area prior to completion of the goal, whereas the infants did not exhibit such predictive tendencies (see S1 Text and S1B Fig).

We investigated the spatial distribution of fixations to the actor’s face area relative to the total time looking towards the three areas combined. A 3 (phase) × 2 (action type) × 2 (group: infants, children) mixed ANOVA revealed a significant main effect of phase (*F*_2,56_ = 10.78, *P* < 0.001, partial *η* ^2^ = 0.28) and a significant interaction between phase and group (*F*_2,56_ = 9.60, *P* < 0.001, partial *η* ^2^ = 0.26). A 3 (phase) × 2 (action type) repeated-measures ANOVA for each group revealed a significant main effect of phase for the children (*F*_2,28_ = 31.07, *P* < 0.001, partial *η* ^2^ = 0.69), whereas no significant difference was evident for the infants (*F*_2,28_ = 2.88, *P* = 0.73, partial *η* ^2^ = 0.17). *Post hoc* testing (Bonferroni adjusted) for the children revealed that the ratio of looking towards the face area in the during-goal phase was significantly lower than in both the before- and after-goal phases for both conditions (all *Ps* < 0.01, Fig 3C; also see S5 and S6 Movies). Thus, just like human adults, children -but not infants- differentially referred to the actor’s face while coding the goal-directedness of the action. These referential looking behaviours were evident regardless of whether the predicted goal was completed. The infants attended proportionally less to the goal area than to the face of the agent.

## General Discussion

The current results demonstrate that chimpanzees and human adults exhibit different face-scanning patterns when observing others’ goal-directed actions. Chimpanzees rarely changed their scanning patterns depending on the sequential progressing of dynamic goal directed actions, regardless of whether the predicted action goal was achieved. Human adults and children, but not infants, differentially referred to the actor’s face depending on the course of the action sequence. Specifically, human adults attended more to the actor’s face *after* confirming that the predicted goal was not achieved. These findings suggest that humans developmentally acquire a predisposition to observe and understand goal-directed actions by integrating the social information available during goal-directed motor actions with cues from actors’ faces.

Previous studies have suggested that chimpanzees and monkeys discriminate between whether goal-directed actions are adequate or inadequate on the basis of their own daily experiences [18–20, 29, 30]. For example, following familiarization with goal-directed actions, monkeys attended to an actor’s face significantly longer when the actor performed motor acts that violated the expected action sequence compared with when the actor performed the predicted motor sequence [19]. However, this observation with monkeys had left unresolved the question regarding exactly *when* they timely scan actors’ faces. Here, we have addressed this issue by focusing on the timing of facial references along with the process of encoding the observed actions in humans and non-human primates. We demonstrated that the timing involved in seeking additional referential information from faces is totally different between humans and chimpanzees.

When and why do human adults pay more attention to an actor’s face, especially when a possible goal is not achieved? Our study does not provide direct evidence regarding the neural or psychological mechanisms that may answer these questions, yet at least one possibility warrants acknowledgment. The current findings suggest that in implausible and/or ambiguous contexts, we need to *explicitly* identify actors’ intentions by making inferences concerning the mental states of other agents independently from our own mental states. We expand on this proposal as follows.

When observing familiar goal directed actions such as juice being poured into a cup, the mirror neuron system is activated which enables us to understand the intentions or goals of others’ actions *implicitly* or automatically. On the other hand however, when we observe implausible or ambiguous actions such as juice being poured on a table, a cognitive mechanism is required that may go beyond the mirror neuron system and implicit understanding which enables us to *explicitly* or actively understand other people’s mental states that might be different from those of our own (e.g., why did the actor pour juice from a bottle onto the table top rather than the glass?). This explicit understanding may require numerous neural systems that are involved in higher-order cognitive functioning related to the self-other distinction by which we actively make inferences concerning the mental states of other agents (i.e., *perspective-taking* and *mentalizing*).

Recent neuroimaging studies of mentalizing using human adults seem to support this ‘explicit’ view [31–34], suggesting that *explicit* mentalizing tasks involve a neural system with the following three components: (1) the medial prefrontal cortex (MPFC), which is most likely the basis of the decoupling mechanism that distinguishes mental state representations from physical state representations; (2) the temporal poles, which are thought to be involved in accessing social knowledge in the form of scripts developed through experience and that record the particular goals and activities that take place in a particular setting at a particular time; and (3) the posterior superior temporal sulcus (STS), which is most likely the basis for the detection of agency. In relation to these findings and our ‘explicit’ proposal, the activation of these neural networks related to mentalizing might be underlying humans’ tendency to look back at faces, especially in ambiguous contexts as was evident in this study. Future imaging research using our experimental paradigm should test this assumption.

The developmental data of Experiment 3 may also verify this explicit assumption. We found that human infants and children exhibited social referencing patterns that differed from those of adults. The 12-month-old infants’ attention towards the actor’s face did not differ either between conditions or across phases. These results are consistent with our previous finding demonstrating that *after* goal achievement, 8- and 12-month-old human infants continued to pay attention to the face after the action goal is achieved whereas adults looked less at the actor’s face [20]. The current study further examined the scanning patterns of 3.5-year-old children and demonstrated that they increased their visual attention towards the actor’s face during the after-goal phase, similar to the adults in the incongruent condition, but the children referred to the actor’s face regardless of whether the predicted goal was completed or not. Interpreting these findings, one possibility is that children are still developing their mentalizing ability and its underlying neural systems [35, 36]. On the other hand, recent developmental studies using an eye tracking methodology have suggested that human infants use ostensive-referential signals such as eye-contact when addressing adults in social learning contexts (e.g., [37, 38]). Accordingly, our child participants perhaps increased their attention towards actor’s faces after both congruent and incongruent actions since they have become aware that faces offer referential signals to learn about such social situations. Whilst these proposal involving mentalizing and ostensive cues are both plausible, it is also possible for them to be complimentary. Indeed, it is important to consider how the information regarding goal-directed motor acts is developmentally integrated with the information obtained from actors' ostensive cues, and how such an early capacity relates to the ability to judge the perspectives of others [39]. We also note that the extent to which chimpanzees have the ability for explicit mentalizing remains controversial [40–42]. The current findings imply that chimpanzees are perhaps poor at mentalizing because they do not use (and integrate) facial cue information with goal-directed perception.

Potential limitations of the current study warrant address. Firstly, the number of chimpanzees participating was relatively small (6 chimpanzees). To ensure that this sample is generally representative of chimpanzees, it is therefore necessary to replicate our findings with a larger sample size. Secondly, although our chimpanzees were quite familiar with humans and human environments, it might be possible that they did not simply attend to the actions of a different species (human) and that perhaps they would if watching videos of chimpanzees. Although our own prior research found no effect of human versus chimpanzee actors [20], further chimpanzee research using conspecifics is worthy of consideration.

Our study emphasizes the importance of simultaneously examining the relationship between time-series changes in face-scanning patterns when coding the goal-directedness of actions. Such an approach is important in bridging prior research from two important topical issues in both developmental and comparative research fields, which may in turn offer additional insights into the neurocognitive basis underlying crucial social abilities such as mentalizing and perspective-taking.

## Supporting Information

S1 Dataset The dataset associated with Experiment 1. (XLSX)

S2 Dataset The dataset associated with Experiment 2. (XLSX)

S3 Dataset The dataset associated with Experiment 3. (XLSX)

S1 Fig Fixation latency on the goal relative to the defined zero point. (a) Latencies to fixate on the cup or container area (i.e., the goal) relative to the onset of pouring juice into the cup (Experiment 1) or transporting the ball into the container (Experiment 2) (defined as the zero point) in human adults and chimpanzees. (b) Latencies to fixate on the cup area (i.e., the goal) relative to the onset of pouring juice into the cup (defined as the zero point) in human adults (Experiment 1), infants, and children (Experiment 3). Positive values correspond to fixation shifts to the cup area prior to the onset of pouring. The error bars represent SEM.
(TIF)

S2 Fig Comparisons of temporal changes in the ratios of participants who were attending to the three AOIs—Face, Trajectory, and Cup (red, green, and blue lines on the main vertical axis, respectively)—and the velocity profiles for the actor’s right hand during the video stimuli (black line on the secondary vertical axis) for the congruent (left) and incongruent (right) actions in Experiment 1; (a) human adults and (b) chimpanzees. Positive velocity values correspond to a rightward direction across the screen.
(TIF)

S1 Movie Movie sample of a congruent test trial in Experiment 1 (human adult vs. chimpanzee adult). (AVI)

S2 Movie Movie sample of an incongruent test trial in Experiment 1 (human adult vs. chimpanzee adult). (AVI)

S3 Movie Movie sample of a congruent test trial in Experiment 2 (human adult vs. chimpanzee adult). (AVI)

S4 Movie Movie sample of an incongruent test trial in Experiment 2 (human adult vs. chimpanzee adult). (AVI)

S5 Movie Movie sample of a congruent test trial in Experiment 3 (infant vs. child). (AVI)

S6 Movie Movie sample of an incongruent test trial in Experiment 3 (infant vs. child). (AVI)

S1 Text Supplementary results for Experiments 1–3. (DOC)
