# Supplementary figures and images for: Humans but Not Chimpanzees Vary Face-Scanning Patterns Depending on Contexts during Action Observation
Source: PLoS One. 2015 Nov 4;10(11):e0139989. doi: 10.1371/journal.pone.0139989 (PMC4633149; doi:10.1371/journal.pone.0139989)

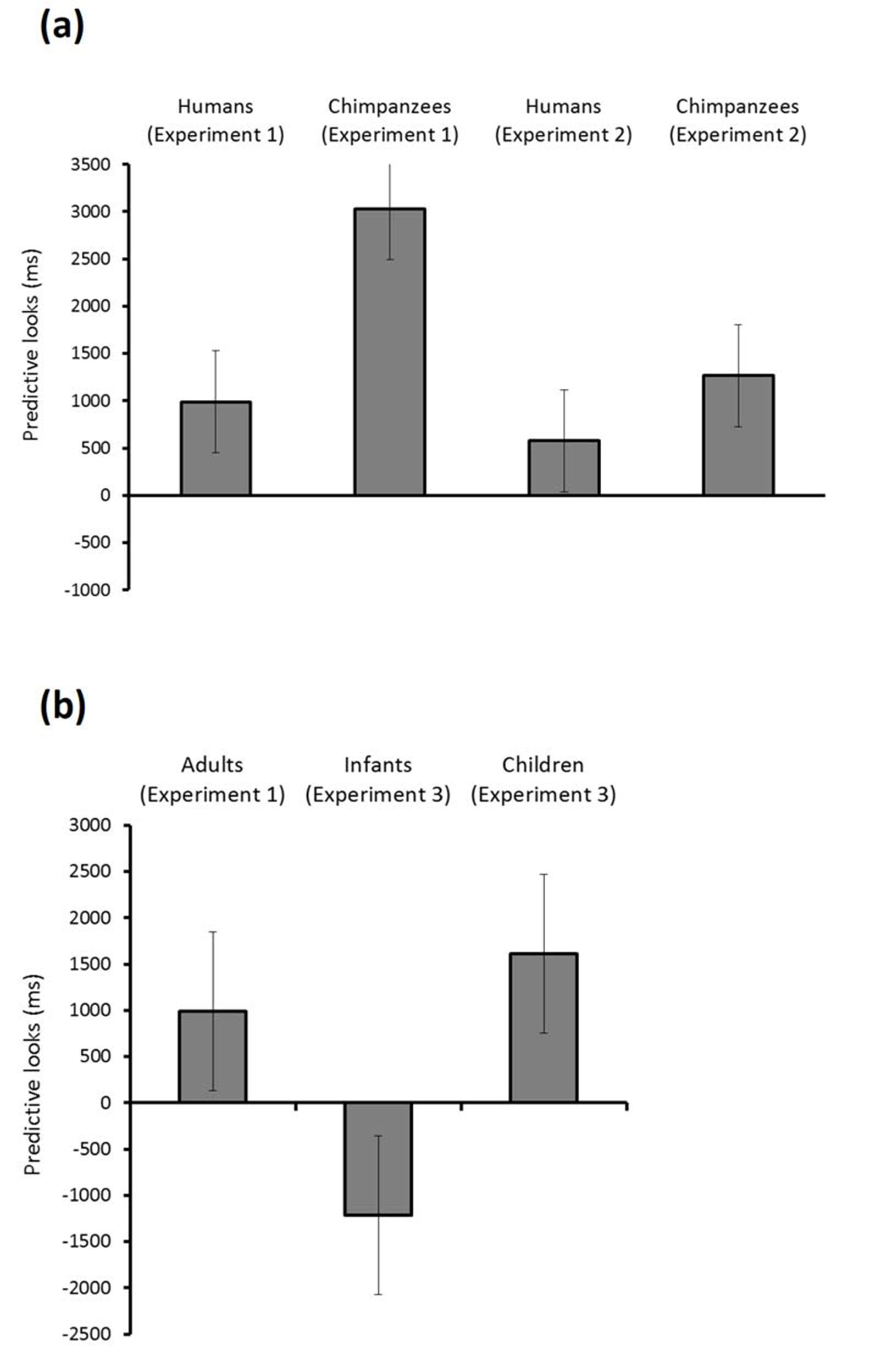

Supplement: S1 Fig — (a) Latencies to fixate on the cup or container area (i.e., the goal) relative to the onset of pouring juice into the cup (Experiment 1) or transporting the ball into the container (Experiment 2) (defined as the zero point) in human adults and chimpanzees. (b) Latencies to fixate on the cup area (i.e., the goal) relative to the onset of pouring juice into the cup (defined as the zero point) in human adults (Experiment 1), infants, and children (Experiment 3). Positive values correspond to fixation shifts to the cup area prior to the onset of pouring. The error bars represent SEM. (TIF) [file pone.0139989.s004.tif]

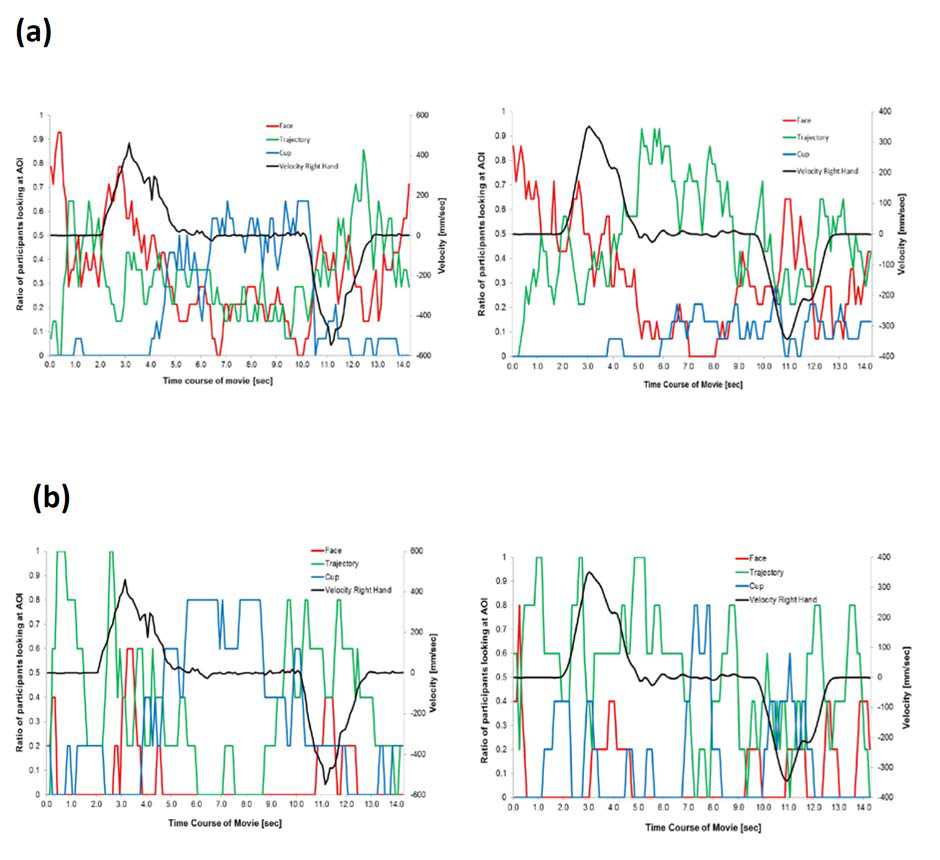

Supplement: S2 Fig — Positive velocity values correspond to a rightward direction across the screen. (TIF) [file pone.0139989.s005.tif]
