# Supplementary material for: Humans but Not Chimpanzees Vary Face-Scanning Patterns Depending on Contexts during Action Observation
Source: PLoS One. 2015 Nov 4;10(11):e0139989. doi: 10.1371/journal.pone.0139989 (PMC4633149; doi:10.1371/journal.pone.0139989)
Supplement: S1 Text — (DOC) [file pone.0139989.s012.doc]

**S1 Text. Supplementary Results for Experiment 1-3.**

**Humans but not chimpanzees vary face-scanning patterns depending on contexts during action observation**

*Masako Myowa-Yamakoshi, Chisato Yoshida, and Satoshi Hirata

*Corresponding author

E-mail: myowa.masako.4x@kyoto-u.ac.jp

**Supplementary results for Experiment 1**

The latencies of the participants’ fixation shifts to the Goal AOI were compared with respect to the onset of pouring juice in the first presentation of the congruent action. If looking at the Goal AOI occurred prior to the onset of pouring the juice into the cup, (defined as a zero point), the trial was considered predictive. Using single-sample t-tests, latency data (in ms) were tested against the zero point to assess whether performance was significantly predictive or reactive. The latency of fixation shifts to the Goal AOI were also compared between the two groups using a one-way ANOVA. Latency data were tested against 0 ms (defined as the onset of pouring juice) to assess whether performance was significantly predictive (positive latencies, ms) or reactive (negative latencies, ms). On average, human adults (mean = 988.57, *t*_13_ = 3.80, *P* < 0.01, Cohen’s *d* = 1.44) and chimpanzees (mean = 3,032.5, *t*_5_ = 5.03, *P* < 0.01, Cohen’s *d* = 2.90) shifted their gaze to the goal prior to the juice being poured into the cup. A comparison of the two groups revealed a significant difference between predictive eye movements to the goal (*F*_1,18_ = 13.58, *P* < 0.01, *η*^2^ = 0.19, S1a Fig).

Next, a one-way ANOVA was conducted to compare the average fixation durations to the test stimuli between the humans and chimpanzees. However, no differences were evident between the chimpanzees and humans (*F*_1,18_ = 1.02, *P* = 0.33, 344 ms for humans and 285 ms for chimpanzees). We also conducted a 2 (area: face, cup + trajectory) × 2 (group: humans, chimpanzees) mixed ANOVA, which yielded no significant differences (area: *F*_1,18_ = 0.29, *P* = 0.60, group: *F*_1,18_ = 3.66, *P* = 0.07, partial *η*^2^ = 0.17, area × group: *F*_1,18_ = 0.98, *P* = 0.34, partial *η*^2^ = 0.05).

To analyse the ratio of the number of fixations on the actor’s face area to the total number of fixations towards the three areas (Face, Trajectory, and Goal AOIs) combined, we conducted a 3 (phase) × 2 (action type) × 2 (group) ANOVA. We observed significant main effects of group (*F*_1,18_ = 15.17, *P* < 0.01, partial *η*^2^ = 0.46) and phase (*F*_2,36_ = 8.79, *P* < 0.01, partial *η*^2^ = 0.33) and a marginally significant two-way interaction between phase and action type (*F*_2,36_ = 3.00, *P* = 0.06, partial *η*^2^ = 0.14). A 3 (phase) × 2 (action type) repeated-measures ANOVA for each group revealed a significant main effect of phase for humans (*F*_2,26_ = 10.51, *P* < 0.01, partial *η*^2^ =0.45). *Post hoc* testing (Bonferroni) for the human group revealed that the number of fixations towards the face area in the before-goal phase was significantly higher than in the during-goal phase for both the congruent and incongruent conditions (*Ps* < 0.05). Only in the congruent condition was number of fixations towards the face area higher in the after-goal phase compared to the during-goal phase (*P* = 0.02). In the chimpanzee group, only for the congruent action was the number of fixations towards the face area in the before-goal phase was significantly higher than in the during-goal phase (*P* < 0.05).

**Supplementary results for Experiment 2**

The mean looking time durations towards the stimuli were 8.0 s (s.d.= 1.4) for the congruent action and 9.0 s (s.d.= 1.2) for the incongruent action for human adults and 5.2 s (s.d. = 1.8) for the congruent action and 4.7 s (s.d.= 2.5) for the incongruent action for chimpanzees. The preliminary analysis revealed significant effects of group in the total fixation durations (*F*_1,18_ = 31.88, *P* <0.001, *η*^2^ = 0.41) and average fixation durations (*F*_1,18_ = 16.95, *P* < 0.01, *η*^2^ = 0.24). The durations were shorter for chimpanzees than for humans (315 ms for humans and 201 ms for chimpanzees). We also conducted a 2 (area: face, cup+trajectory) × 2 (group) mixed ANOVA that yielded significant main effects of area (*F*_1,18_ = 8.18, *P* < 0.05, partial *η*^2^ = 0.31) and group (*F*_1,18_ = 21.04, *P* = 0.001, partial *η*^2^ = 0.54) and a significant interaction between area and phase (*F*_1,18_ = 8.74, *P* < 0.01, partial *η*^2^ = 0.33). In particular, humans attended to the face area when they viewed the object-related actions of other individuals.

The latencies of the participants’ fixation shifts to the Goal AOI were compared to the onset of transporting the first grasped ball during the first presentation of the congruent action. On average, both human adults (mean = 575.00, *t*_13_ = 6.79, *P* < 0.001, Cohen’s *d* = 2.57) and chimpanzees (mean = 1,265.83, *t*_5_ = 5.91, *P* < 0.01, Cohen’s *d* = 3.41) shifted their gaze to the goal prior to the ball being placed into the container. A comparison between the two groups revealed a significant effect of predictive eye movements to the goal (*F*_1,18_ = 13.46, *P* < 0.01, *η*^2^ = 0.19; S1a Fig.).

The ratio of the number of fixations on the actor’s face area was examined using a 3 (phase) × 2 (action type) × 2 (group) ANOVA, revealing significant main effects of group (*F*_1,18_ =43.00, *P* < 0.001, partial *η*^2^ = 0.71) and phase (*F*_2,36_ = 5.02, *P* < 0.03, partial *η*^2^ = 0.22). A 3 (phase) × 2 (action type) repeated-measures ANOVA for each group revealed a significant main effect of phase (*F*_2,26_ = 6.62, *P* < 0.01, partial *η*^2^ =0.34) and a significant interaction between phase and action type in humans (*F*_2,26_ = 4.45, *P* < 0.03, partial *η*^2^ =0.26) but not in chimpanzees (phase: *F*_2,10_ = 2.49, *P* = 0.13, partial *η*^2^ =0.33, phase × action type: *F*_2,10_ = 1.53, *P* = 0.27, partial *η*^2^ =0.23). *Post hoc* testing (Bonferroni) for the human group revealed that the ratio of the number of fixations towards the face area was higher in the after-goal phase compared to both the during-goal and before-goal phases but only in the incongruent action (*Ps* < 0.01). No significant differences were evident for the chimpanzee group.

**Supplementary results for Experiment 3**

The mean durations of looking time towards the stimuli were 8.1 s (s.d.= 1.6) for the congruent action and 6.0 s (s.d.= 1.8) for the incongruent action for the 12-month-old infants and 9.5 s (s.d.= 1.6) for the congruent action and 7.9 s (s.d.= 2.8) for the incongruent action for the 3.5-year-old children. The preliminary analysis of the total fixation durations revealed a significant effect of group (*F*_1,28_ = 6.82, *P* < 0.02, *η*^2^ = 0.04). However, no significant group differences were evident for the average fixation durations towards the test stimuli (*F*_1,28_ = 2.12, *P* = 0.16, *η*^2^ = 0.01; 325 ms for infants and 376 ms for children). We also conducted a 2 (area: face, cup+trajectory) × 2 (group) mixed ANOVA that yielded no significant differences (area: *F*_1,28_ = 3.72, *P* = 0.06, group: *F*_1,28_ = 1.08, *P* = 0.31, area × group: *F*_1,28_ = 0.10, *P* = 0.75).

The latencies of the participants’ fixation shifts to the Goal AOI were compared to the onset of pouring juice during the first presentation of the congruent action. On average, the children (mean = 1,612.33, *t*_14_ = 4.00, *P* < 0.01, Cohen’s *d* = 1.46) but not the infants (mean = -1,214.67, *t*_14_ = 1.52, *P* = 0.15) shifted their gaze to the goal prior to the juice being poured into the cup. A comparison between the two groups revealed a significant effect of predictive eye movements to the goal (*F*_1,28_ = 9.96, *P* < 0.01, *η*^2^ = 0.07; S1b Fig.).

The ratio of the number of fixations towards the face area was examined using a 3 (phase) × 2 (action type) × 2 (group) ANOVA. A significant main effect of phase was evident (*F*_2,56_ = 6.34, *P* < 0.01, partial *η*^2^ = 0.19), and a significant interaction between phase and group (*F*_2,56_ = 6.34, *P* < 0.05, partial *η*^2^ = 0.19) was also present. A 3 (phase) × 2 (action type) repeated-measures ANOVA for each group revealed a significant main effect of phase in the children (*F*_2,28_ = 21.72, *P* < 0.001, partial *η*^2^ = 0.61) but not in the infants (*F*_2,28_ = 1.61, *P* = 0.22, partial *η*^2^ = 0.10). *Post hoc* testing (Bonferroni) for the children’s group revealed that the number of fixations towards the face area was lower in the during-goal phase compared to both the before- and after-goal phases (all *Ps* < 0.01). No significant differences were evident for the infant group.
